# Cold Exposure Induces Proliferation of Mature Brown Adipocyte in a ß3-Adrenergic Receptor-Mediated Pathway

**DOI:** 10.1371/journal.pone.0166579

**Published:** 2016-11-15

**Authors:** Keigo Fukano, Yuko Okamatsu-Ogura, Ayumi Tsubota, Junko Nio-Kobayashi, Kazuhiro Kimura

**Affiliations:** 1 Department of Biomedical Sciences, Graduate School of Veterinary Medicine, Hokkaido University, Sapporo 060–0818, Japan; 2 Laboratory of Histology and Cytology, Graduate School of Medicine, Hokkaido University, Sapporo 065–0013, Japan; Brown University Warren Alpert Medical School, UNITED STATES

## Abstract

Hyperplasia of brown adipose tissue (BAT) is a fundamental mechanism for adaptation to survive in the cold environment in rodents. To determine which cell types comprising BAT contribute to tissue hyperplasia, immunohistochemical analysis using a proliferative marker Ki67 was performed on the BAT from 6-week-old C57BL/6J mice housed at 23°C (control) or 10°C (cold) for 5 days. Interestingly, in the control group, the cell proliferative marker Ki67 was detected in the nuclei of uncoupling protein 1-positive mature brown adipocytes (7.2% ± 0.4% of brown adipocyte), as well as in the non-adipocyte stromal-vascular (SV) cells (19.6% ± 2.3% of SV cells), which include preadiopocytes. The percentage of Ki67-positive brown adipocytes increased to 25.6% ± 1.8% at Day 1 after cold exposure and was significantly higher than the non-cold acclimated control until Day 5 (21.8% ± 1.7%). On the other hand, the percentage of Ki67-positive SV cells gradually increased by a cold exposure and peaked to 42.1% ± 8.3% at Day 5. Injection of a ß3-adrenergic receptor (ß3-AR) agonist for continuous 5 days increased the number of Ki67-positive brown adipocytes even at Day 1 but not that of SV cells. In addition, the ß3-AR antagonist, but not ß1-AR antagonist, attenuated the cold exposure-induced increase in the number of Ki67-positive brown adipocytes. These results suggest that mature brown adipocytes proliferate immediately after cold exposure in a ß3-AR-mediated pathway. Thus, proliferation of mature brown adipocytes as well as preadipocytes in SV cells may contribute to cold exposure-induced BAT hyperplasia.

## Introduction

The function of brown adipose tissue (BAT) is to transfer energy into heat through the thermogenic activity of mitochondrial uncoupling protein 1 (UCP1), which dissipates the proton gradient that is normally used to drive the synthesis of cellular ATP [1–3]. BAT thermogenesis is important for the maintenance of body temperature in cold circumstances, particularly in small rodents [4]. In the cold environment, brown adipocytes are activated by the sympathetic nerve system: norepinephrine (NE) released from nerve endings activates the ß-adrenergic receptor (ß-AR) on brown adipocytes to induce lipolysis. Liberated fatty acids activate UCP1 and are simultaneously used as substrates for thermogenesis.

In both humans and rodents, cold exposure induces the hyperplasia of BAT and increases the whole-body thermogenic capacity [5–7]. BAT is composed of several types of cells, including UCP1-expressing mature brown adipocytes and non-adipocytes stromal-vascular (SV) cells, which consist of preadipocytes, interstitial cells, and vascular endothelial cells [5]. It is suggested that preadipocytes are responsible for BAT hyperplasia, although a few days of cold stimulation increases thymidine incorporation into both adipocytes and SV cells [5,8]. Moreover, BAT hyperplasia is suppressed by the denervation of sympathetic fibers to BAT [9] and is reproduced by an injection of NE [10], indicating that it is controlled by the sympathetic nervous system. It is likely that ß1-AR, which is expressed in both preadipocytes and mature brown adipocytes [11], is mainly involved in cold-induced hyperplasia *in vivo* [12] and preadipocyte proliferation *in vitro* [10, 13]. Thus, the proliferation of preadipocytes and their subsequent differentiation may significantly contribute to the increased number of brown adipocytes. On the other hand, it is suggested that cell proliferation is enhanced by the stimulation of ß3-AR, which is exclusively expressed in mature adipocytes [14]. However, there is little information on the proliferation of mature brown adipocytes and their contribution to BAT hyperplasia.

To determine the role of mature adipocyte proliferation in BAT hyperplasia, immunohistochemical analysis using a proliferative marker Ki67 on BAT from cold acclimated mice was performed. We found that cold exposure induced the early onset of proliferation of mature brown adipocytes in a β3-AR-mediated pathway, and subsequently the proliferation of SV cells in a β1-AR-mediated pathway.

## Materials and Methods

### Materials

Rabbit anti-serum against UCP1 was a gift from Dr. Teruo Kawada (Kyoto University; Kyoto, Japan). Antibodies against p44/p42 MAPK (ERK) and the phosphorylated form of histone H3 and ERK were purchased from Cell Signaling Technology (Beverly, MA, USA). Antibodies against proliferating cell nuclear antigen (PCNA) were purchased from Santa Cruz Biotechnology (Santa Cruz, CA, USA), whereas antibodies against ß-actin were from Sigma-Aldrich (St. Louis, MO, USA). Antibodies against Ki67 and MCT1 were from Abcam (Cambridge, MA, USA) and Merck Millipore (Billerica, MA, USA), respectively. Anti-rabbit secondary antibodies conjugated with Alexa Fluor 488 or Alexa Fluor 594 were obtained from Thermo Fisher Scientific (Gaithersburg, MD, USA), and anti-chicken secondary antibody conjugated with Alexa Fluor 594 was obtained from Thermo Scientific (Rockford, IL, USA).

### Animals

The experimental procedures and care of animals were approved by the Animal Care and Use Committee of Hokkaido University (Hokkaido, Japan). All experiments using mice were conducted in the animal facility approved by the Association for Assessment and Accreditation of Laboratory Animal Care (AAALAC) International. C57BL/6J mice were purchased from Japan SLC Inc. (Shizuoka, Japan). Mice were housed in plastic cages within an air-conditioned room at 23°C with a 12:12-h light:dark cycle and given free access to laboratory chow (Oriental Yeast; Tokyo, Japan) and tap water. Animal health condition was monitored daily during the experiments. For the cold exposure experiment, each mouse was housed in a plastic cage placed in a cold room at 10°C for 1–5 days. Other mice were injected subcutaneously with the ß3-AR agonist CL316,243 (CL, 0.1 mg/kg, once a day; Sigma) for 1–5 days. For experiments using ß-AR antagonists, mice were injected subcutaneously with the ß1-AR antagonist metoprolol (5 mg/kg, twice a day; Sigma) or the ß3-AR antagonist SR59230A (1 mg/kg, twice a day; Sigma), and housed in a cold room at 10°C for 1 day. During the experiments, no animals became ill or died. Mice were euthanized with carbon dioxide, and interscapular BAT was promptly removed and weighed. Tissue specimens were fixed in 4% paraformaldehyde for histological examinations or transferred into liquid nitrogen for Western blot analysis and the measurement of DNA content.

### Preparation of mature adipocytes and SV cells

SV cells and mature adipocytes were isolated as previously reported [15]. Briefly, interscapular BAT and perigonadal white adipose tissue (WAT) were minced into small pieces and digested in Krebs-Ringer HEPES buffer containing fatty acid-free bovine serum albumin (BSA, 10 mg/ml), 2.5 mM glucose, and collagenase (1 mg/ml) for 1 h at 37°C with shaking at 90 cycles/min. The cell suspension was passed through a 200-μm nylon filter, and the filtrate was centrifuged at 200 ×g for 2 min at room temperature. The floating cells were collected as mature adipocytes. The pellet was suspended and passed through a 25-μm nylon filter, centrifuged at 200 ×g for 2 min at room temperature, and the resultant pellet was used as SV cells. Mature adipocytes and SV cells were washed three times with Krebs-Ringer HEPES buffer to eliminate collagenase and BSA. The collected cells were used for Western blot analysis, which was performed as previously reported [15].

### Immunofluorescent staining of BAT sections

For immunofluorescent staining, deparaffinized sections at 5-μm thickness were incubated in 0.3% hydrogen peroxide in methanol. For the detection of Ki67, microwave-enhanced antigen retrieval was performed by boiling the slides in 10 mM citrate buffer (pH 6.0) containing 0.05% Tween 20 for 10 min. After washing in phosphate-buffered saline, the slides were incubated for 1 h with 10% normal goat serum then with the primary antibodies (rabbit anti-Ki67, 1:300; or rabbit anti-UCP1, 1:200; together with chicken anti-MCT-1, 1:200) overnight at 4°C, followed by incubation with fluorescence-conjugated second antibody (1:200) for 1 h. After washing, the sections were stained with 4′,6-diamidino-2-phenylindole (DAPI) and mounted with ProLong Gold Antifade (Life Technologies).

### DNA content

DNA content was assessed using a fluorometric method with bisbenzimidazole (Hoechst no.33258), as described previously [16]. In brief, tissue specimens were homogenized in Tris-EDTA buffer (10 mM Tris and 1 mM EDTA, pH7.4), and incubated with bisbenzimidazole in the 50 mM phosphate buffer containing 2 M NaCl and 2 mM EDTA. The fluorescence intensity was measured using Microplate Fluorometer (Fluroroskan Ascent; Thermo Fisher Scientific) with 360 nm excitation filter and 460 nm emission filter. The DNA concentration of each sample was estimated by comparison with a standard curve using salmon sperm DNA.

### Data analysis

Values are expressed as mean ± standard error of the mean (SE). Statistical analyses were performed using Student’s *t*-test or analysis of variance followed by Scheffe’s post-hoc test.

## Results

### Identification of cell types expressing proliferative marker Ki67 in the BAT

**Fig 1:**
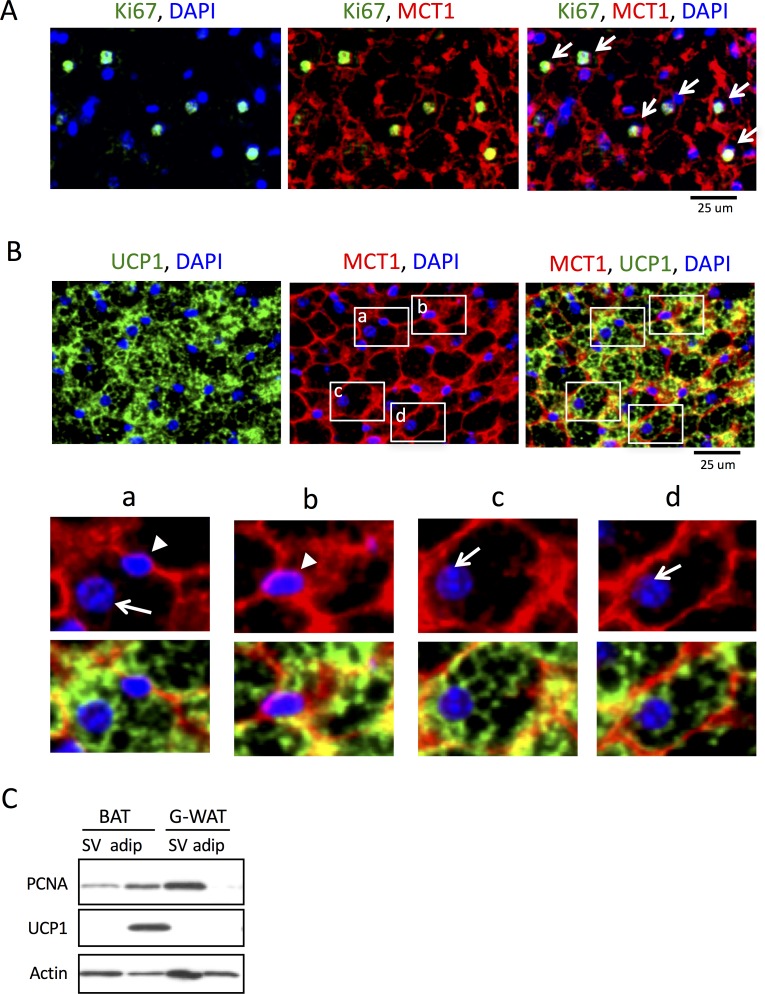
Proliferating cell markers, Ki67 and PCNA, are detected in both mature brown adipocytes and SV cells in BAT. (A) Immunofluorescent staining of DAPI (blue), MCT1 (red), and UCP1 (green) enable two types of cells in the BAT sections to be distinguished: mature brown adipocytes expressing UCP1 in the cytoplasm with round nuclei isolated from the MCT-positive cell membrane (arrows in a, c, and d), and SV cells with flat-shaped nuclei distributed between the MCT1-positive cell membranes (arrowheads in a and b). (B) Dual immunostaining for Ki67 (green) and MCT1 (red) shows that the proliferating cell marker is present in the nuclei of mature brown adipocytes (arrows). (C) Western blot analysis reveals that PCNA is expressed in both SV cells and mature brown adipocytes (brown) separated from BAT, whereas it is detected only in SV cells from gonadal white adipose tissue (G-WAT).

To analyze the cell types in the BAT specimens, we used antibodies against UCP1, which is exclusively expressed in the mitochondria of brown adipocytes, and monocarboxylate transporter 1 (MCT1), which is selectively expressed in the plasma membrane of brown adipocytes [17]. Dual immunostaining with these antibodies showed membrane-specific and cytosolic localization of MCT1 and UCP1, respectively (Fig 1A). In the double-stained cells, nuclei stained with DAPI were large and round-shaped and dissociated from MCT1-positive membrane, and they were identified as brown adipocytes (arrows in Fig 1Aa, c, d); however, small and flat-shaped nuclei distributed between MCT1-positive cells were also observed (arrowheads in Fig 1Aa, b). Because the latter cells possessing narrow cytoplasm lacked in any UCP1 signal, we defined them as non-adipocyte SV cells.

To examine cell proliferation in the BAT, we performed immunofluoresent staining using specific antibodies against a cell proliferation marker Ki67, together with MCT1. In the BAT from non-cold-acclimated control mice, certain number of Ki67-positive cells were present (Fig 1B, left). Based on the abovementioned criteria, most Ki67-positive cells observed in the BAT were identified as brown adipocytes with MCT1 immunoreactivity on cell membrane (arrows in Fig 1B, right). These results suggest that certain numbers of UCP1-expressing mature brown adipocytes were in a proliferative stage. To confirm the proliferation of the brown adipocytes, they were separated from the SV fraction and analyzed by Western blotting. UCP1 was detected only in the mature adipocyte fraction of the BAT, whereas PCNA was found in both fractions (Fig 1C). Because the PCNA level in the mature adipocyte fraction was higher than that in the SV fraction, it was unlikely that the PCNA detected in the mature adipocyte fraction was due to contamination of SV cells in this fraction. In contrast to the BAT, PCNA was only detected in the SV fraction of the gonadal WAT.

### Cold exposure induces an early increase in the number of proliferating brown adipocytes

**Fig 2:**
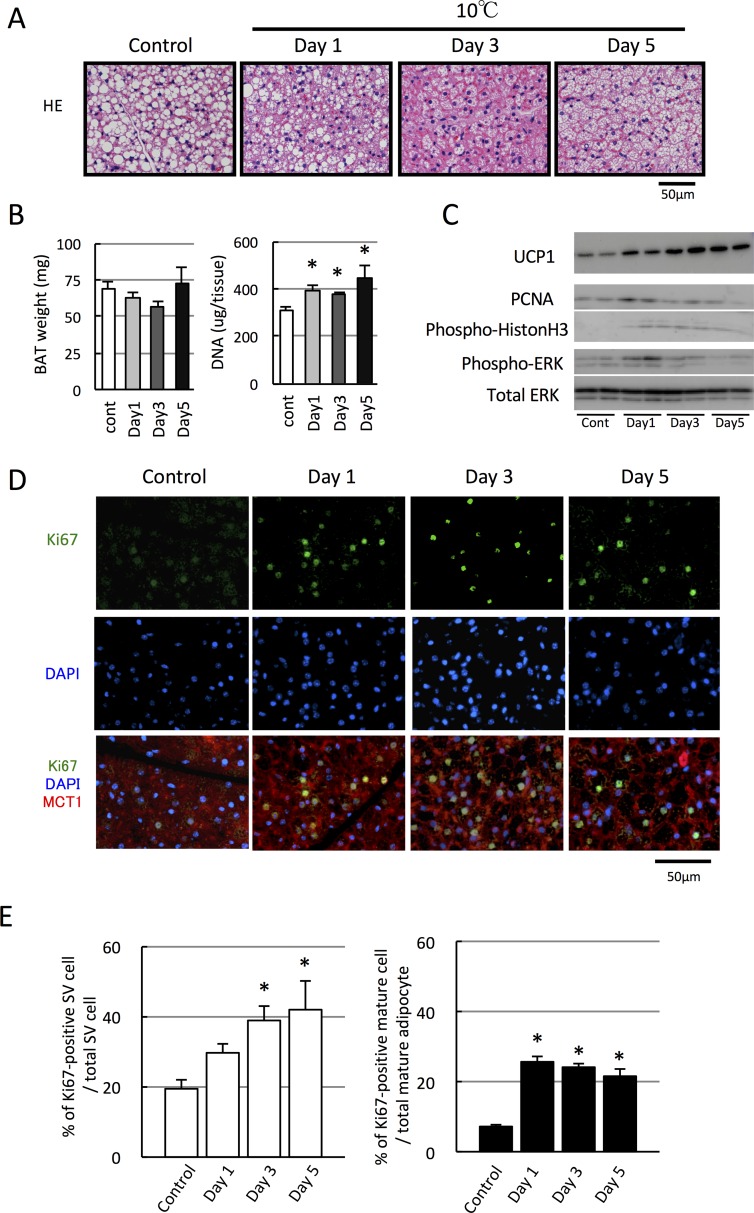
Cold exposure increases the number of proliferating brown adipocytes and proliferating SV cells. Mice were acclimated to cold temperature (10°C) for 1–5 days. (A) Sections from BAT of each mouse were stained with hematoxylin and eosin (H&E). (B) DNA content in BAT, but not tissue weight, increased after Day 1 (n = 4, *p < 0.05 vs. control without cold acclimation). (C) Expression of PCNA and UCP1 proteins as well as the phosphorylated forms of histone H3 and ERK also increased after cold exposure. (D, E) Representative images of BAT sections stained with Ki67, MCT1, and DAPI (E). Quantified graphs showing that an increased number of Ki67-positive cells in both brown adipocytes and SV cells after the cold exposure compared to the non-cold exposed control group (n = 4, *p < 0.05).

It is well documented that hyperplasia of BAT occurs in rodents exposed to a cold environment. In this study, when mice were acclimated to a mildly cold temperature (10°C) for 5 days, DNA content in BAT increased from Day 1 to Day 5, although BAT weight tended to decrease until Day 3, accompanied with a decrease in size of intracellular lipid droplets (Fig 2A and 2B). Consistently, the expression of cell proliferation markers such as PCNA and phosphorylated histone H3 and ERK were increased, particularly on Day 1 (Fig 2C). Moreover, the number of Ki67-positive cells markedly increased after Day 1 (Fig 2D). These results clearly indicate that BAT hyperplasia occurs even 1 day after the onset of cold exposure.

The percentage of Ki67-positive cells in BAT was approximately 6% before cold acclimation (control) and increased approximately 19%–24% after cold acclimation. To determine which cells, brown adipocytes or SV cells, possess Ki67-signals, they were separately counted based on the aforementioned criteria (Fig 2E). The percentage of Ki67-positive brown adipocytes in total brown adipocyte was 7.2% ± 0.4% in the control group and increased to 25.6% ± 1.8% on Day 1. Although it decreased thereafter, it was still significantly higher than that in the control group, even at Day 5 (21.8% ± 1.7%). On the other hand, the number of Ki67-positive SV cells in total SV cells was 19.6% ± 2.3% in the control group and gradually increasing after Day 1 and peaking to 42.1% ± 8.3% at Day 5. These results indicate that mild cold exposure triggers the proliferation of both brown adipocytes and SV cells, albeit with different time courses, and that the proliferation of brown adipocytes is most likely to be involved in the early phase of cold-induced hyperplasia of BAT.

To confirm that the brown adipocytes and SV cells are going through the S phase of cell cycle in response to cold exposure, we conducted EdU incorporation assay. On Day 1 after the cold exposure, the EdU-incorporated cell was observed although their number was lower than that of Ki67-positive cell (S1 Fig). However, both EdU-positive brown adipocytes and SV cells were observed, suggesting that they actually proliferate.

### ß3-adrenergic stimulation selectively increases the number of proliferating brown adipocytes

**Fig 3:**
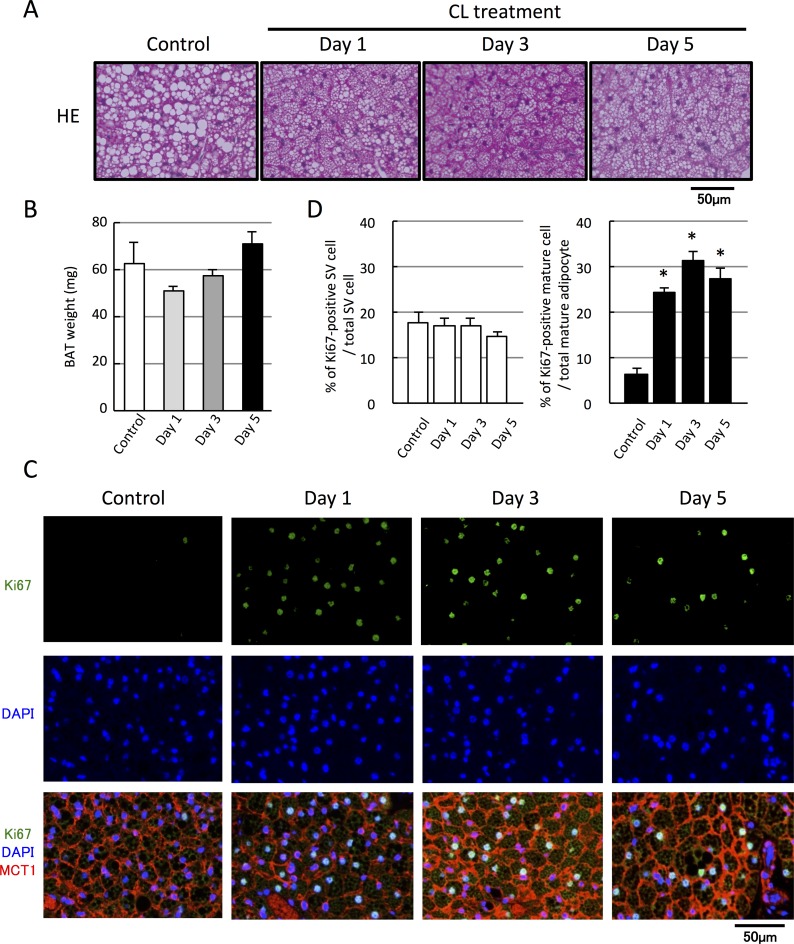
β3-adrenergic stimulation increases the number of proliferating brown adipocytes, but not of proliferating SV cells. Mice were injected with the β3-adrenergic agonist CL316,243 (CL; 0.1 mg/kg, s.c., once a day) for 1–5 days. (A) BAT sections from each mouse were stained with hematoxylin and eosin (H&E). (B) Tissue weight of BAT tends to decrease at Day1. (C) Representative images of BAT sections stained with Ki67 antibody together with MCT1 and DAPI (D). Quantified graphs showing that the number of Ki67-positive brown adipocytes, but not that of SV cells, increased from Day 1 after CL treatment (C, n = 4, *p <0 .05 vs. non-treated control).

To determine the involvement of the sympathetic nervous system in cold-induced enhancement of brown adipocyte proliferation, mice were administered the ß3-AR-specific agonist, CL316,243 (CL; 0.1 mg/kg, once a day) for 5 days. Because CL treatment activates BAT thermogenesis [12], the size of intracellular lipid droplets became small from Day 1 after the treatment (Fig 3A), accompanied by a decrease in tissue weight (Fig 3B). Moreover, the number of Ki67-positive cells was markedly increased by CL treatment (Fig 3C), and the percentage of Ki67-positive brown adipocytes increased from 6.5% ± 1.2% to 24.4% ± 1.1% by Day 1 and was maintained at this level until Day 5 (27.6% ± 2.2%) (Fig 3D). However, CL treatment failed to increase the number of Ki67-positive SV cells (Fig 3D). These results indicate that ß3-AR stimulation leads to an increase in the number of proliferating brown adipocytes but not that of proliferating SV cells.

### Cold-induced brown adipocyte proliferation is mediated by the β3-adrenergic receptor

**Fig 4:**
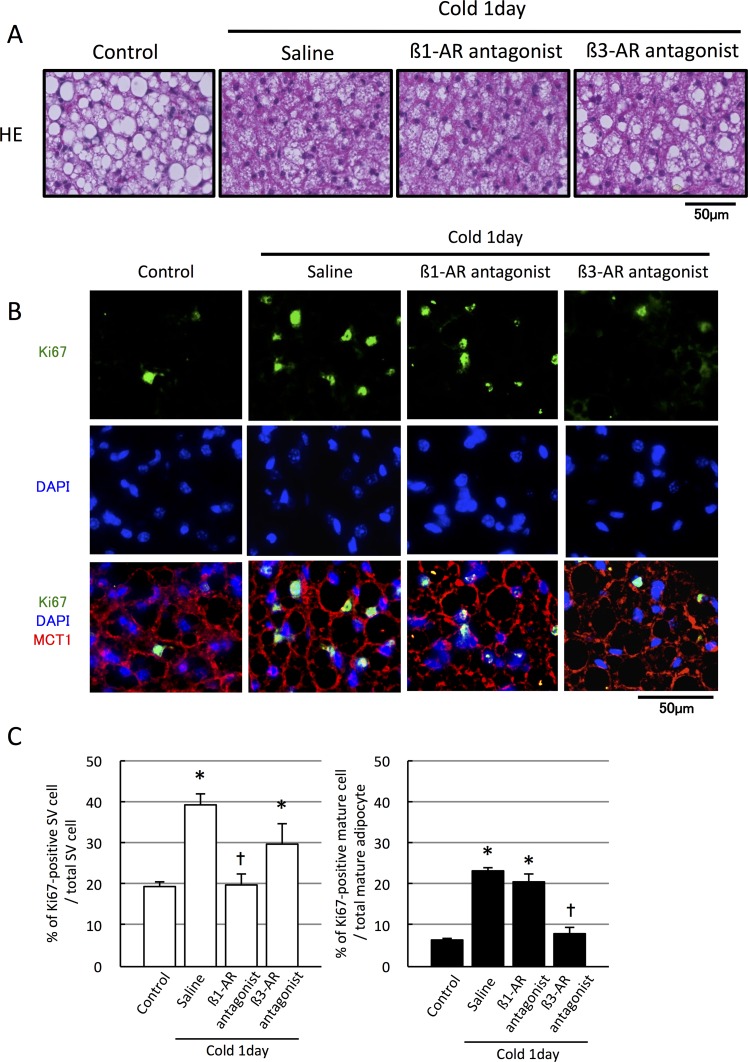
Inhibition of ß3-adrenergic receptor reduces the number of proliferating mature brown adipocytes during cold exposure. Mice injected subcutaneously with saline, a ß1-AR antagonist (metoprolol, 5 mg/kg, twice a day), or ß3-AR antagonist (SR59230A, 1 mg/kg, twice a day), were acclimated to cold temperature (10°C) for 24 h. (A) BAT sections from each mouse were stained with hematoxylin and eosin (HE). (B, C) Representative images of BAT sections stained with Ki67 antibody together with MCT1 and DAPI. (C) Quantification failed to show an increase in the number of Ki67-positive brown adipocytes after cold acclimation in the presence of the ß3-AR antagonist, whereas an increase was observed in the presence of the ß1-AR antagonist (n = 3, *p < 0.05 vs. non-cold acclimated control, †p < 0.05 vs. cold-acclimated with saline injection group).

To confirm the involvement of the sympathetic nervous system in the cold-induced enhancement of brown adipocyte proliferation, mice were injected with the ß1-AR or ß3-AR antagonists and then exposed to cold temperatures (10°C) for 24 h. Cold exposure decreased the size of intracellular lipid droplet in saline- and ß1-AR antagonist-injected mice but not in ß3-AR antagonist-injected mice (Fig 4A), suggesting selective inhibition of thermogenesis by the ß3-AR antagonist. Moreover, the cold exposure-induced increase in the number of Ki67-positive mature brown adipocyte was clearly blocked by the ß3-AR antagonist but not by the ß1-AR antagonist (Fig 4B and 4C). In contrast, proliferation of SV cells induced by cold exposure was blocked by the ß1-AR antagonist but not by the ß3-AR antagonist (Fig 4B and 4C).

## Discussion

In the present study, we demonstrated that the cell proliferation marker Ki67 was detected on UCP1-positive brown adipocytes in control mice and that the number of Ki67-positive brown adipocytes markedly increased 1 day after cold exposure, whereas the increase in non-adipocyte SV cells followed a much slower time course. We also found that injection of a ß3-AR agonist selectively increased the number of Ki67-positive brown adipocytes and that the cold exposure-induced increase in the number of Ki67-positive brown adipocytes was attenuated by the inhibition of ß3-AR, but not that of ß1-AR. These findings suggest that mature brown adipocytes have the ability to proliferate upon the activation of ß3-AR and possibly contribute to BAT hyperplasia by cold exposure-induced activation of the sympathetic nervous system.

Cold exposure is known to induce the proliferation of preadipocytes and vascular endothelial cells [7–9, 18] in BAT. Of these, preadipocytes differentiate into mature brown adipocytes, resulting in the enhancement of BAT function and BAT hyperplasia. On the other hand, it is most likely that the increase in the number of endothelial cells reflects the active angiogenesis in response to the cold exposure and contributes to the enhancement of BAT function [19]. Consistent with these reports, we found that the number of Ki67-positive SV cells, including preadipocytes and vascular endothelial cells, gradually increased from Day 1 to Day 5 after the onset of cold exposure. The proliferation of SV cells seems to be mediated by ß1-AR, and these results are consistent with the previous observation *in vivo* [12] and *in vitro* [10, 13]. In contrast, we found a marked increase in the number of Ki67-positive brown adipocytes expressing UCP1 at Day 1 of cold exposure, and proliferation of brown adipocytes was controlled by a ß3-AR-mediated pathway.

Based on the results described above, it appears that the proliferation of mature brown adipocytes contributes to the early phase of BAT hyperplasia, whereas the proliferation of SV cells contributes to its later phase. It is to be noted that the number of EdU-positive cell was much lower than that of Ki67-positive cell. It may be reasonable because Ki67 is a marker of cells which have entered into cell cycle and expressed during all the course, whereas EdU is incorporated to DNA during S phase. However, considering that as much as 40% of SV cells and more than 20% of brown adipocytes were positive for Ki67 after cold acclimation whereas the DNA content increased by 1.4-fold, it is plausible that not all of the Ki67-positive cell actually dividing even though, although they are in the proliferative state.

The reason for the difference in the time course of proliferation between brown adipocytes and SV cells is still unknown; however, it is likely that the proliferation of SV cells may not be directly regulated by NE. Indeed, cold stimulation induces the expression of several growth factors such as IGF1 [20, 21], VEGF-A [22–24], NGF [25, 26], and FGF-2 [27, 28], which induce the proliferation of several types of cells in SV fraction [29]. Thus, these downstream growth factors may contribute to the proliferation of SV cells, thereby resulting in a longer time needed to stimulate the proliferation in SV cells. On the other hand, NE directly stimulates the proliferation of mature brown adipocytes via ß3-AR-mediated pathway. Further investigation is required to elucidate the factors that induce the proliferation of each cell type.

The degree of the contribution of each cell types to the cold exposure-induced BAT hyperplasia is not clear. To estimate their contribution, the composition of mature adipocytes and SV cells in the tissue is needed to be clarified. In this study, the percentage of the mature adipocytes was as much as 76.9% ± 0.6% of total cells observed in the BAT sections in the control group. However, the ratio may not reflect the actual cellular composition in the whole tissue because the cellular size of mature brown adipocyte is much larger than that of SV cells, resulting in the over-estimation of the mature brown adipocyte number. Further study is required to clarify the precise mechanism and the contribution of each cell types to the BAT hyperplasia.

In conclusion, we demonstrate that brown adipocytes retain their proliferative ability even after differentiation and that this unique feature of brown adipocytes is involved in BAT hyperplasia induced by cold exposure. Because BAT contributes to the regulation of body fat through its energy-dissipating activity and is a therapeutic target for the treatment of obesity [2, 3], our findings may provide new insights into the development of agents that control BAT amount.

## Supporting Information

S1 Fig (TIFF)
